# A deployable curriculum with 3D printed skills trainers for altered airway management

**DOI:** 10.1186/s12909-023-05013-6

**Published:** 2024-01-08

**Authors:** Madison V. Epperson, Arushi Mahajan, Rishabh Sethia, Nolan Seim, Kyle VanKoevering, Robert J. Morrison

**Affiliations:** 1 Department of Otolaryngology-Head & Neck Surgery, University of Michigan, 1500 E. Medical Center Drive, Ann Arbor, MI 48109-4241 USA; 2 University of Michigan Medical School, Ann Arbor, MI USA; 3 Department of Otolaryngology-Head & Neck Surgery, The Ohio State University, Columbus, OH USA

**Keywords:** Tracheostomy, Laryngectomy, Airway curriculum, Simulation, Task trainer, 3D-printed

## Abstract

**Background:**

Altered Airway Anatomy (AAA), including tracheostomies and laryngectomies, may represent an area of unease for non-Otolaryngology trainees, due to a lack of exposure, structured education, or dedicated training in altered airway management. Inability to effectively stabilize an altered airway is associated with significant risk of patient morbidity and mortality. This study aims to assess the efficacy of a concise curriculum using three-dimensional (3D) printed airway models for skill training in improving Anesthesiology trainees’ competency in AAA management.

**Methods:**

A prospective cohort of 42 anesthesiology residents at a tertiary care institution were guided through a 75-min curriculum on AAA, including case discussion, surgical video, and hands-on practice with tracheostomy and laryngectomy skills trainers. Pre- and post- course surveys assessing provider confidence (Likert scale) and knowledge (multiple choice questions) were administered. Additionally, an observed skills competency assessment was performed.

**Results:**

Self-perceived confidence improved from a summative score across all domains of 23.65/40 pre-course to 36.39/40 post-course (*n* = 31, *p* < 0.001). Technical knowledge on multiple choice questions improved from 71 to 95% (*n* = 29, *p* < 0.001). In the completed skills competency assessment, 42/42 residents completed 5/5 assessed tasks successfully, demonstrating objective skills-based competency.

**Conclusions:**

This study demonstrates an improvement in anesthesiology resident self-assessed confidence, objective knowledge, and skills based competency surrounding management of patients with AAA following a 75-min simulation-based curriculum.

**Supplementary Information:**

The online version contains supplementary material available at 10.1186/s12909-023-05013-6.

## Background

Altered airway anatomy (AAA), inclusive of patients who have undergone tracheostomy or laryngectomy, is commonly seen in both the inpatient and perioperative setting. These procedures are performed for a variety of indications such as acute or chronic respiratory failure, upper airway obstruction, including head and neck cancer, or complications of treatment of head and neck cancer, such as a non-functional larynx [1–3]. Prevalence is difficult to determine due to the wide range of etiologies, but for respiratory failure alone, there was an estimated annual incidence of 28.4–39.7 tracheostomies per 100,000 adults in the United States from 2002–2017 [4].

Despite significant anatomic differences and resultant airway management strategies between tracheostomy and laryngectomy patients, less than 5% of frontline providers can describe the anatomic differences, and only 41% administered oxygen via the correct route in laryngectomy patients [5]. Various resources and consensus statements have been created surrounding tracheostomy and laryngectomy education for providers and patients to ameliorate the uncertainty in management of altered airways [3, 6–8]. Nevertheless, these resources are often under-utilized, and providers still cite discomfort with AAA. This is particularly the case with non-Otolaryngology providers, due to infrequent exposure to this patient population and lack of dedicated education or training in altered airway management [9, 10]. However, non-Otolaryngology providers such as those in the discipline of Anesthesiology, Emergency Medicine, or Critical Care are often the first responders and must act to stabilize the airway before specialty surgical providers arrive.

Unfortunately, complications related to altered airways continue to be associated with significant morbidity and mortality [7, 11]. Tracheostomy-related adverse events account for up to 50% of airway-related deaths and hypoxic brain injury within critical care units [11]. Evidence has shown that a protocolized and multidisciplinary approach to tracheostomy care decreases morbidity and mortality [12–14]. Additionally, hands-on simulation airway training has been shown to improve provider confidence and skill with airway related tasks [15–17]. With this in mind, we endeavored to develop a readily portable and deployable curriculum to improve competency in AAA knowledge and management for non-surgical providers. We leveraged fidelity of 3D printing to produce a life-like but low cost skills trainer to supplement the curriculum. We sought to evaluate the effect of the curriculum and trainer on anesthesiology resident perceived confidence, knowledge, and skills competency.

## Methods

**Fig. 1:**
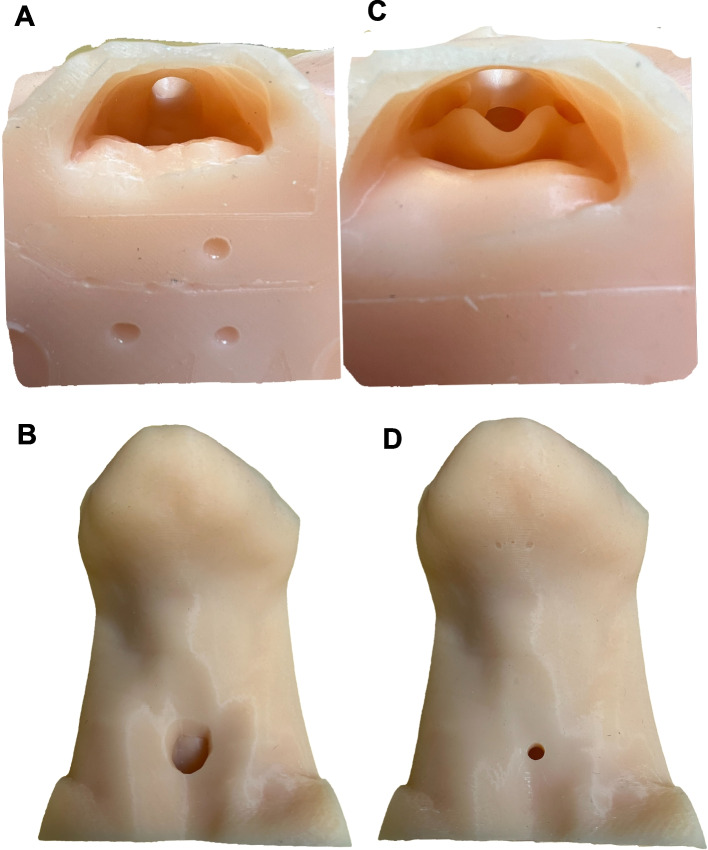
Laryngectomy (**A**, **B**) and Tracheostomy (**C**, **D**) three-dimensional printed airway models from superior (**A**, **C**) and anterior (**B**, **D**) views

Institutional review board approval was obtained from the University (HUM00066405). This curriculum was modified from a prior validated AAA curriculum used for third-year medical students to better meet the needs of a resident cohort [16]. An observed skills-based competency assessment was added to quantitatively measure procedural knowledge. Two Otolaryngology providers served as the instructors for three sessions, with 12–15 anesthesiology residents per session. The sessions were performed at a tertiary care institution. Residents were guided through a 75-min simulation-based curriculum on AAA including case discussions, a video on laryngectomy anatomy, and hands-on practice with 3D-printed tracheostomy and laryngectomy airway models (Fig. 1) with various types of pediatric and adult tracheostomy tubes, endotracheal tubes, laryngectomy tubes, caps, and passy-muir valves. The learning objectives are listed in Table 1. The 3D-printed tracheostomy and laryngectomy models were created using the Medical Modeling, Materials, and Manufacturing Lab (M4 Lab) at The Ohio State University and were based off 3D models segmented from a CT of the neck of a normal adult male and an adult male who has previously undergone laryngectomy.

**Table 1:** Learning objectives

1. I can identify a patient with a tracheostomy and have a basic understanding of the airway anatomy in patients with a tracheostomy tube
2. I can identify a patient with a laryngectomy and have a basic understanding of the airway anatomy in patients with a laryngectomy
3. I understand problems that can occur with altered airway anatomy and steps to stabilize the patient, including mucus plugs, false passages, and airway bleeding
4. Performance Assessment- I can describe and assemble the components of tracheostomy tube, and I am able to successfully replace a tracheostomy tube
5. Performance Assessment- I can successfully place a Blomsinger (laryngectomy tube) and understand next steps if a laryngectomy patient needs assisted ventilation

Learning objectives including both knowledge objectives and skilled tasks that participants should be readily able to complete for the altered airway anatomy course

The curriculum included a large group discussion on AAA, with a focus on indications for a tracheostomy and laryngectomy, anatomical differences between the two (with a surgical laryngectomy video), and differences in management (15 min). This was followed by four case-based discussions on mucus plugs, false passages, airway bleeding, and a laryngectomy patient needing positive pressure ventilation (20 min). The group was split into two smaller groups for the final portion of the session: the hands-on portion with the airway models (40 min). The instructor first presented the various types of pediatric and adult tracheostomy tubes, endotracheal tubes, laryngectomy tubes, caps, and passy-muir valves (including when caps vs. passy muir valves are used). Further teaching included a demonstration of the steps of a tracheostomy tube exchange with the tracheostomy model and illustrated options for establishing positive pressure ventilation in the laryngectomy model.

Pre- and post- course surveys assessing provider confidence and knowledge were administered. Questions aimed to assess basic knowledge of tracheostomy and laryngectomy anatomy, how to replace and confirm placement of a tracheostomy tube (suction catheter, capnography, air flow, flexible tracheoscopy), how to deliver positive pressure ventilation to a laryngectomy patient, how to identify and manage a false passage or mucus plug, and first steps to manage bleeding into the airway. The full question batteries are included in Additional file 1.

The provider confidence survey assessed confidence via a 5-point Likert type scale, with a score of 5 indicating the participant strongly agreed they understood or could complete the task (relative to the question) and a score of 1 indicating they strongly disagreed. A composite score was generated from the 8 individual items by summing the score for each question to create a single numeric score (maximum of 40). The knowledge assessment survey was a series of 5 multiple choice questions and 1 short answer. A percent correct was calculated. A observed skills competency assessment was performed following the session (Table 2). This assessment entailed an Otolaryngology provider assessing each individual perform the five skills listed in Table 2. The skill was marked as either performed as “not done,” “done incompletely or incorrectly,” or “ done successfully.”

**Table 2:** Items assessed on skills competency assessment

1. Can learner identify an inner cannula, obturator, pilot balloon, and outer cannula?
2. Can learner assemble and successfully place a tracheostomy tube?
3. Can learner suction a tracheostomy with a flexible suction catheter?
4. Can learner place a laryngectomy tube?
5. Can learner establish ventilation if a laryngectomy patient needs positive pressure ventilation? (Options include placing cuffed trach or endotracheal tube in stoma)

Items assessed on the skills competency assessment at the conclusion of the course

Statistical analysis was performed using the Wilcoxon Signed Rank test to compare pre- and post-course perceived confidence and knowledge, with a *p* value of 0.05 set as level of statistical significance. For the objective skills assessment, the number of skills out of five done successfully was recorded. Qualitative data was collected in the post-course assessment as to areas the course could be improved.

## Results

### Perceived confidence in AAA management

**Fig. 2:**
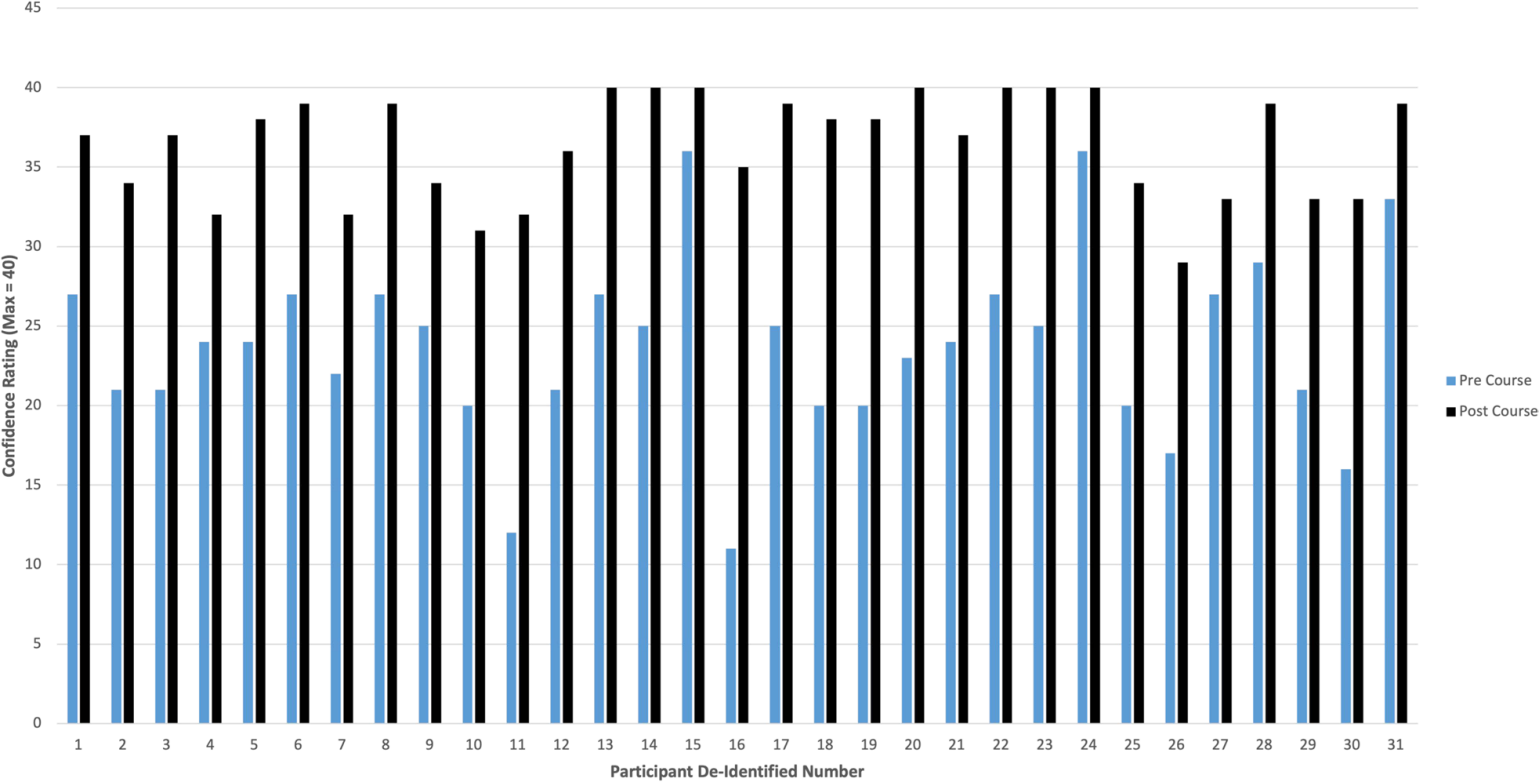
Perceived Pre- and Post- Course confidence in altered airway management assessed via eight Likert Scale questions (1–5, 1 = strongly disagree, 5 = strongly agree). Individual (*n* = 31) summative scores (maximum 40) displayed with pre-course scores indicated by light blue and post-course by dark blue. Significantly improved confidence post-course (*p* value =  < 0.001)

Thirty one individuals completed pre and post course perceived confidence surveys. A Wilcoxon Signed Rank test was used for statistical analysis. Self-perceived confidence improved from a summative score across all domains of 23.65/40 pre-course to 36.39/40 post-course (*n* = 31, *p* < 0.001). Individual summative pre- and post- course confidence are illustrated in Fig. 2.

### Objective knowledge in AAA management

**Fig. 3:**
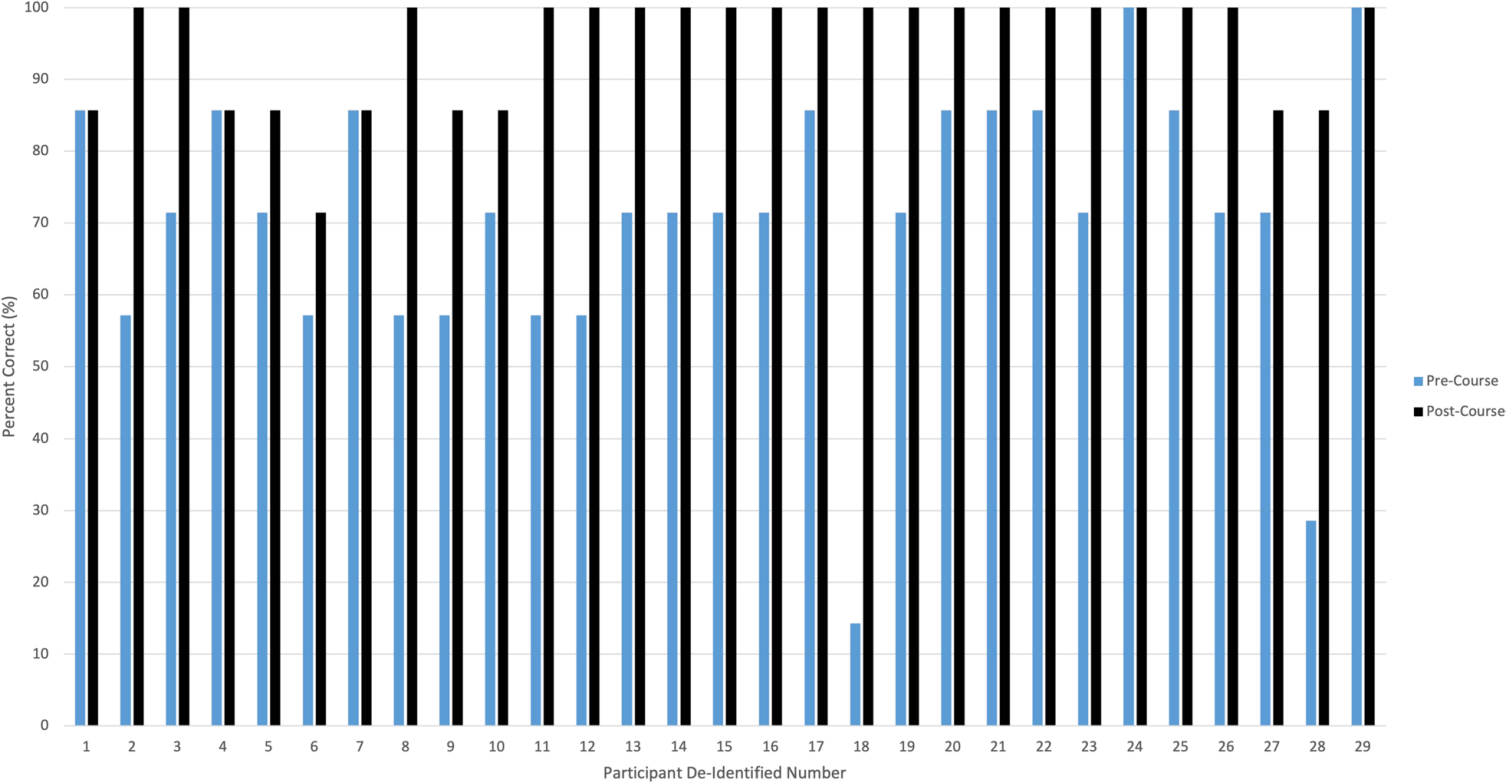
Perceived Pre- and Post- Course knowledge assessment in altered airway management assessed via five multiple choice questions. Individual (*n* = 29) percent correct (%) displayed with pre-course scores indicated by light blue and post-course by dark blue. Significantly improved knowledge post-course (*p* value =  < 0.001)

Twenty nine individuals completed pre and post course knowledge assessment multiple choice questions. A Wilcoxon Signed Rank test was used for statistical analysis. Technical knowledge improved from 71 to 95% (*n* = 29, *p* < 0.001). Individual pre- and post- course percent correct are illustrated in Fig. 3.

### Skills-based competency in AAA management

Forty two individuals completed the post-course skills competency assessment, 42/42 learners completed 5/5 assessed tasks successfully, as assessed by Otolaryngology providers. Note, the second cohort was not verbally reminded to complete the post course confidence and knowledge assessment, explaining the discrepancy between the number of individuals completing the post-course written confidence and knowledge assessment compared to the observed skills assessment.

## Discussion

Despite the high incidence of morbidity and mortality surrounding management of altered airways, providers that interface with tracheostomies and laryngectomies still cite significant discomfort, prompting the need for targeted interventions to increase provider competency and improve patient safety. In this study, we sought to determine the efficacy of a concise AAA curriculum utilizing 3D printed tracheostomy and laryngectomy skill trainers in improving Anesthesiology resident perceived confidence, knowledge, and skills competency. We noted an improvement in self-assessed confidence, objective knowledge, and skills based competency following the 75-min simulation-based curriculum.

The role of simulation in graduate medical education is well established [18]. 3D printing is increasingly being utilized to augment training for a broad array of applications within this realm such as within urologic, neurosurgical, and vascular residency programs, due to the ability to manufacture low-cost, high-fidelity 3D structures for education and simulation [19–21]. The 3D printed tracheostomy and laryngectomy airway task trainers have previously been utilized in a curriculum designed for medical students, but have not been used for skills development or assessment in physicians or providers actually caring for AAA patients [16]. Despite the studies that have assessed various simulation-based AAA curriculums for different cohorts, this study is the largest to date assessing the efficacy of a concise curriculum incorporating 3D printed airway skill trainers for an anesthesiology resident cohort.

Implementation and validation of a cost-effective and concise curriculum for anesthesiology residents is essential as these providers regularly interface with AAA, often serving as the first provider responsible for stabilizing critical airways. In this study, we found that anesthesiology residents lacked confidence and technical knowledge addressing common problems arising with altered airways such as mucus plugs, false passages, and upper airway bleeding, which can be fatal if not recognized early and managed expeditiously [22]. After a 75 -minute course, residents had significantly improved confidence and knowledge with objective evidence of their competency in performing various basic airway skills such as a tracheostomy change or establishing positive pressure ventilation in a laryngectomy patient. The task trainer augments the curriculum as it allows hands-on practice and objective assessment of competency while remaining low cost and widely deployable.

When placing our findings in the context of previous studies, similar to other student and resident cohorts, dedicated curriculums surrounding the altered airway prove beneficial in terms of both perceived confidence and knowledge [9, 15–17, 23]. Differing curriculum structures have been employed, both virtual and in-person, with varying degrees of didactics, case-based simulation, and hands-on training for various cohorts including medical students, pediatric, internal medicine, emergency medicine, anesthesiology, and otolaryngology residents. Mehta et. al evaluated a 60-min session for tracheostomy care and management including both didactics and case-based simulation in a resident group consisting of emergency medicine, internal medicine, and intensive care unit providers. They found improved comfort level and knowledge post-course, noting some specialty specific differences [9]. As different specialties have different daily exposures to AAA, tailoring curricula to specific specialties may prove important. For example, airway curriculums may look different for surgical vs. non-surgical cohorts. Nguyen et. al developed a curriculum for Otolaryngology residents focusing on surgical airways and pediatric intubation in cadaver, mannequin, and animal models, noting improved technical skills [15]. Emphasis on a different skill set is important in non-surgical cohorts. Only one study, by Davis et. al, has assessed an AAA curriculum with anesthesiology residents [17]. They looked at a cohort of emergency medicine, internal medicine, and anesthesia residents, with *n* = 14 anesthesia residents. There was variation by group, but overall, they had improved self-assessed confidence scores, knowledge scores, and observed tracheostomy tube change proficiency post-course. Their model was a high-fidelity simulation mannequin. Our results support those reported by Davis et al. in a larger cohort of anesthesia residents, utilizing smaller, more portable and low-cost models manufactured using 3D printing.

Our results must be interpreted in the context of the limitations of our study. One limitation is that not all individuals completed the post-course perceived confidence survey or technical knowledge survey. We presume these individuals may have simply forgotten to or did not wish to complete the post-course assessment, but their reason for failing to complete these surveys is unknown, and therefore, their post-session confidence and knowledge cannot be accounted for. Nevertheless, all individuals completed the objective skills competency assessment, and all individuals performed all tasks correctly. Therefore, at least proficient demonstration of a skill set was noted within this group. Additionally, we did not perform a pre-course observed skills competency assessment. This was considered, but ultimately not included, as the thought of having individuals attempt to perform skills they are less familiar with at the beginning of the session may be somewhat discouraging and may not be the best use of limited time. The long term impact of this curriculum on competency is unknown, and there is the possibility for skills, confidence, and knowledge attrition. A six month or year follow up to assess retention of skills, knowledge, and confidence would also be beneficial. We plan to follow up with a survey for our established cohort. Of note, similar simulation-based courses have demonstrated retention [17]. Lastly, while this study validated the efficacy within the anesthesiology resident cohort, adaptive modifications may be necessary if used for other cohorts such as Emergency Medicine or Intensive Care Unit providers. Nevertheless, this curriculum and the 3D printed airway skill trainers provide a foundation for further curriculum development.

## Conclusions

This study demonstrates an improvement in Anesthesiology resident self-assessed confidence, objective knowledge, and skills based competency surrounding management of patients with AAA following a 75-min simulation-based curriculum with 3D printed airway models. This validates the positive impact of this curriculum on both subjective and objective performance in an anesthesiology resident cohort. Comparing adverse events related to altered airways prior to completion of the curriculum to adverse events occurring after the curriculum could serve to further demonstrate the direct impact on patient care. Future directions may be aimed at expanding the curriculum to additional cohorts that may interface with altered airways, which may include but are not limited to Emergency Medicine residents or providers in the Intensive Care Unit. Longitudinal studies to assure retention are warranted. Regarding the task trainer, future models may consider incorporating a false passage space with the understanding that this may increase the complexity and thus, the cost of the model, but would provide increased hands-on experience addressing common complications.

### Supplementary Information

**Additional file 1.** Course surveys and assessments. Includes full pre and post course surveys and knowledge assessment questions as well as the formal skills competency assessment.

## Data Availability

The datasets used during the current study are available from the corresponding author on reasonable request.

## Abbreviation

AAA: Altered Airway Anatomy
