# Supplementary material for: A deployable curriculum with 3D printed skills trainers for altered airway management
Source: BMC Med Educ. 2024 Jan 8;24:39. doi: 10.1186/s12909-023-05013-6 (PMC10773045; doi:10.1186/s12909-023-05013-6)
Supplement: Supplementary file 1 — Additional file 1. Course surveys and assessments. Includes full pre and post course surveys and knowledge assessment questions as well as the formal skills competency assessment. [file 12909_2023_5013_MOESM1_ESM.docx]

**Altered Airway Anatomy Pre-Course Survey**

Thank you for your participation in this study. Please remember that completion of this survey is voluntary and you may withdraw at any time without penalty. However, we appreciate your feedback in order for us to better evaluate the course. Your responses to the following questions will remain confidential. Your anonymous responses may be used for research purposes without your additional informed consent. There is a small risk of breach of confidentiality, but we have taken all the appropriate measures to prevent this. There will be no direct benefit to the participant. If you have any questions, concerns, or complaints about the study, please contact principal investigator Robert Morrison at morrisor@med.umich.edu.

Please answer the questions below to the best of your ability using the following scale:

1 = Strongly Disagree

2 = Disagree

3 = Neutral

4 = Agree

5 = Strongly Agree

*Altered airway anatomy refers to patients who have undergone procedures such as tracheostomy or total laryngectomy to modify their airway.*

1. I can identify a patient with a trach. ____

2. I can identify a patient with a laryngectomy and differentiate them from a ____

tracheostomy patient.

3. I understand the parts of a tracheostomy tube (inner cannula, obturator, faceplate, cuff) ____

and how this is different from a laryngectomy tube (Blomsinger). ____

4. I feel comfortable changing a tracheostomy tube. ____

5. I know what to do if a patient has a mucus plug. ____

6. I know what do if a laryngectomy patient needs positive pressure ventilation. ____

7. I feel comfortable with my ability to independently stabilize AAA patients ____

until expert airway providers arrive.

Mark your experience with AAA with an X on the line.


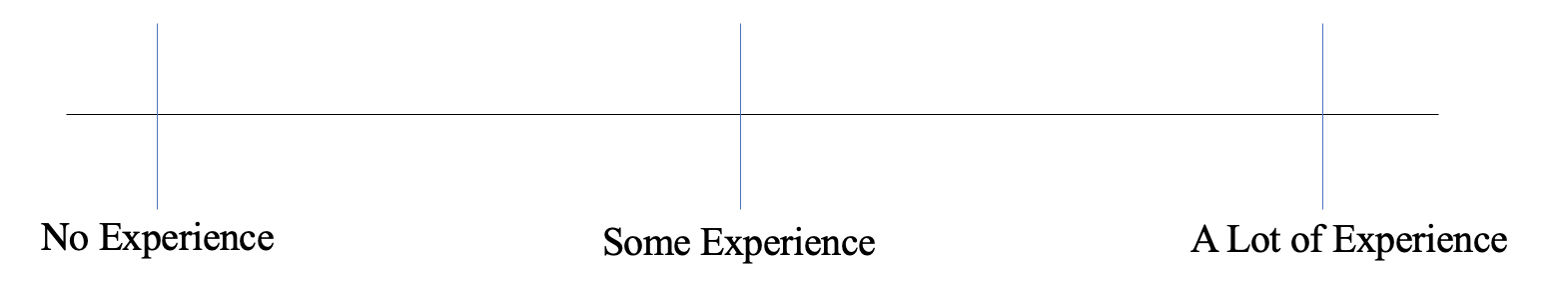


**Altered Airway Anatomy-- Knowledge Assessment (Pre- Course)**

Circle the correct answer choice.

1. Which of these is the best option to place in a tracheostoma for a patient requiring positive pressure ventilation?
   1. Stoma Vent
   2. Cuffless Tracheostomy Tube
   3. Cuffed Tracheostomy Tube
   4. Fenestrated Tracheostomy Tube
   5. Voice Prosthesis
2. What is the name of the item that is used to aid in insertion of the tracheostomy tube into the stoma?
   1. Inner Cannula
   2. Obturator
   3. Outer Cannula
   4. Pilot Balloon
   5. Trach Plug
3. In a patient with a total laryngectomy in respiratory distress, what can be used to ventilate the patient (circle all correct possible answers)?
   1. Bag mask ventilation over mouth and nose
   2. Endotracheal tube through stoma
   3. Transoral intubation
   4. Transnasal intubation
   5. Cuffed tracheostomy tube through stoma
4. You are the first to arrive to a patient with a cuffed tracheostomy tube in place that develops acute high-volume bleeding from their mouth, what is the immediate next best step after calling for help?
   1. Inflate the tracheostomy tube cuff
   2. Remove the tracheostomy tube
   3. Perform transoral intubation
   4. Replace with a cuffless tracheostomy tube
   5. Replace with an endotracheal tube through the stoma
5. What is a method to confirm that a tracheostomy tube is patent and in proper position (circle all correct possible answers)?
   1. Passing a suction catheter easily through the tube
   2. Capnography
   3. Appreciating air flow through the tube
   4. Flexible endoscopy
6. Short Answer-- Name two possible causes of inability to pass a suction catheter in a patient with a tracheostomy tube?
   1. ___________________________________________
   2. ___________________________________________

**Altered Airway Anatomy Post-Course Survey**

Thank you for your participation in this study. Please remember that completion of this survey is voluntary and you may withdraw at any time without penalty. However, we appreciate your feedback in order for us to better evaluate the course. Your responses to the following questions will remain confidential. Your anonymous responses may be used for research purposes without your additional informed consent. There is a small risk of breach of confidentiality, but we have taken all the appropriate measures to prevent this. There will be no direct benefit to the participant. If you have any questions, concerns, or complaints about the study, please contact principal investigator Robert Morrison at morrisor@med.umich.edu.

Please answer the questions below to the best of your ability using the following scale:

1 = Strongly Disagree

2 = Disagree

3 = Neutral

4 = Agree

5 = Strongly Agree

*Altered airway anatomy refers to patients who have undergone procedures such as tracheostomy or total laryngectomy to modify their airway.*

1. I can identify a patient with a trach. ____

2. I can identify a patient with a laryngectomy and differentiate them from a

tracheostomy patient. ____

3. I understand the parts of a tracheostomy tube (inner cannula, obturator, faceplate, cuff) ____

and how this is different from a laryngectomy tube (Blomsinger).

4. I understand how a tracheostomy tube is different than a laryngectomy tube. ____ ____

4. I feel comfortable changing a tracheostomy tube. ____

5. I know what to do if a patient has a mucus plug. ____

6. I know what do if a laryngectomy patient needs positive pressure ventilation. ____

7. I feel comfortable with my ability to independently stabilize AAA patients ____

until expert airway providers arrive.

We really appreciate any positive or critical feedback to improve this curriculum. Please provide any feedback below.

____________________________________________________________________________________________________________________________________________________________

**Altered Airway Anatomy-- Knowledge Assessment (Post-Course)**

Circle the correct answer choice.

1. Which of these is the best option to place in a tracheostoma for a patient requiring positive pressure ventilation?
   1. Stoma Vent
   2. Cuffless Tracheostomy Tube
   3. Cuffed Tracheostomy Tube
   4. Fenestrated Tracheostomy Tube
   5. Voice Prosthesis
2. What is the name of the item that is used to aid in insertion of the tracheostomy tube into the stoma?
   1. Inner Cannula
   2. Obturator
   3. Outer Cannula
   4. Pilot Balloon
   5. Trach Plug
3. In a patient with a total laryngectomy in respiratory distress, what can be used to ventilate the patient (circle all correct possible answers)?
   1. Bag mask ventilation over mouth and nose
   2. Endotracheal tube through stoma
   3. Transoral intubation
   4. Transnasal intubation
   5. Cuffed tracheostomy tube through stoma
4. You are the first to arrive to a patient with a cuffed tracheostomy tube in place that develops acute high-volume bleeding from their mouth, what is the immediate next best step after calling for help?
   1. Inflate the tracheostomy tube cuff
   2. Remove the tracheostomy tube
   3. Perform transoral intubation
   4. Replace with a cuffless tracheostomy tube
   5. Replace with an endotracheal tube through the stoma
5. What is a method to confirm that a tracheostomy tube is patent and in proper position (circle all correct possible answers)?
   1. Passing a suction catheter easily through the tube
   2. Capnography
   3. Appreciating air flow through the tube
   4. Flexible endoscopy
6. Short Answer-- Name two possible causes of inability to pass a suction catheter in a patient with a tracheostomy tube?
   1. ___________________________________________
   2. ___________________________________________

**Skills Competency Assessment**

1. Can learner identify an inner cannula, obturator, pilot balloon, and outer cannula?

___ Not Done

___ Done Incompletely/Incorrectly

___ Done Successfully

1. Can learner assemble and successfully place a tracheostomy tube?

___ Not Done

___ Done Incompletely/Incorrectly

___ Done Successfully

1. Can learner suction a tracheostomy-- appropriate depth (no more than 10cm, finger on suction while pulling out.)

___ Not Done

___ Done Incompletely/Incorrectly

___ Done Successfully

1. Can learner place a laryngectomy tube?

___ Not Done

___ Done Incompletely/Incorrectly

___ Done Successfully

1. Can learner establish ventilation if a laryngectomy patient needs positive pressure ventilation?

(Place either tracheostomy tube or endotracheal tube in stoma and inflate cuff—if ETT is placed, it should only be inserted just past the cuff as to not mainstem the patient)

___ Not Done

___ Done Incompletely/Incorrectly

___ Done Successfully
